# Supplementary material for: Associated factors and socio-economic inequality in the prevalence of thinness and stunting among adolescent boys and girls in Uttar Pradesh and Bihar, India
Source: PLoS One. 2021 Feb 24;16(2):e0247526. doi: 10.1371/journal.pone.0247526 (PMC7904204; doi:10.1371/journal.pone.0247526)
Supplement: Attachment — Submitted filename: PONE-D-20-33031_reviewer.pdf [file pone.0247526.s002.pdf]

PLOS ONE

Associated factors and socio-economic inequality in the prevalence of thinness and stunting among adolescent boys and girls in Uttar Pradesh and Bihar, India

--Manuscript Draft--

**Manuscript Number:**	PONE-D-20-33031
**Article Type:**	Research Article
**Full Title:**	Associated factors and socio-economic inequality in the prevalence of thinness and stunting among adolescent boys and girls in Uttar Pradesh and Bihar, India
**Short Title:**	Thinness, stunting, an co-existence of both among adolescents in two socio-economic backward states in India
**Corresponding Author:**	Strong P Marbaniang<br>International Institute for Population Sciences<br>Mumbai, INDIA
**Keywords:**	Stunting; Thinness; Adolescents; Socio-economic inequality; Decomposition analysis
**Abstract:**	Background<br><br>Despite economic growth observed in developing countries, under-nutrition still continues to be a major health problem. Undernutrition in adolescence can disrupt normal growth and puberty development and may have long-term impact. Therefore, it is important to study the undernutrition among adolescents. This study aimed to assess the prevalence and the associated factors of stunting, thinness and the coexistence of both (stunting and thinness) among the adolescent belonging to Uttar Pradesh and Bihar, India.<br><br>Methods<br><br>The study utilized data from Understanding the Lives of Adolescents and Young Adults (UDAYA) project survey, which was conducted in two Indian states Uttar Pradesh and Bihar, in 2016 by Population Council under the guidance of Ministry of Health and Family Welfare, Government of India. Utilizing information on 20,594 adolescents aged 10-19 years (adolescent boys-5,969 and adolescent girls-14,625), the study examined three outcome variables, i.e., thinness, stunting, and co-existence of both. The study used descriptive and bivariate analysis. Furthermore, the study examined income-related inequality in stunting and thinness through concentration index. At last, the study used Wagstaff decomposition analysis to decompose the concentration index.<br><br>Results<br><br>The prevalence of thinness was higher among adolescent boys as compared to girls (25.8% vs. 13.1%). However, stunting was more prevalent among girls (25.6%) than in boys (39.3%). The odds of stunting and thinness were higher among late adolescents, uneducated adolescents, adolescents not working, and poorest adolescents. Media exposure and wealth index contributed significantly to the inequality in the prevalence of thinness among adolescents, whereas, wealth index was the only significant contributor to the inequality in the prevalence of stunting among adolescents.<br><br>Conclusion<br><br>The study provides an understanding that stunting and thinness is a significant public health concern among adolescents, and there is a need to tackle the issue comprehensively. By tackling the issue comprehensively, we mean that the state government of Uttar Pradesh and Bihar shall screen, assess, and monitor the nutritional status of adolescent boys and girls. The interventions shall focus towards both boys as well as girl adolescents, and particular emphasis should be given to adolescents who belonged to poor households. Also, efforts should be taken by stakeholders to increase family wealth status.

Powered by Editorial Manager® and ProduXion Manager® from Aries Systems Corporation

Order of Authors:	Pradeep Kumar<br>Shobhit Srivastava<br>Shekhar Chauhan<br>Ratna Patel<br>Strong P Marbaniang<br>Preeti Dhillon
Additional Information:	
Question	Response
**Financial Disclosure**<br><br>Enter a financial disclosure statement that describes the sources of funding for the work included in this submission. Review the submission guidelines for detailed requirements. View published research articles from *PLOS ONE* for specific examples.<br><br>This statement is required for submission and **will appear in the published article** if the submission is accepted. Please make sure it is accurate.<br><br>**Unfunded studies**<br>Enter: *The author(s) received no specific funding for this work.*<br><br>**Funded studies**<br>Enter a statement with the following details:<br>• Initials of the authors who received each award<br>• Grant numbers awarded to each author<br>• The full name of each funder<br>• URL of each funder website<br>• Did the sponsors or funders play any role in the study design, data collection and analysis, decision to publish, or preparation of the manuscript?<br>• **NO** - Include this sentence at the end of your statement: *The funders had no role in study design, data collection and analysis, decision to publish, or preparation of the manuscript.*<br>• **YES** - Specify the role(s) played.<br><br>* typeset	The author(s) received no specific funding for this work.
**Competing Interests**<br><br>Use the instructions below to enter a	The authors have declared that no competing interests exist.

competing interest statement for this submission. On behalf of all authors, disclose any competing interests that could be perceived to bias this work—acknowledging all financial support and any other relevant financial or non-financial competing interests.<br><br>This statement **will appear in the published article** if the submission is accepted. Please make sure it is accurate. View published research articles from *PLOS ONE* for specific examples.<br><br>**NO authors have competing interests**<br><br>Enter: *The authors have declared that no competing interests exist.*<br><br>**Authors with competing interests**<br><br>Enter competing interest details beginning with this statement:<br><br>*I have read the journal's policy and the authors of this manuscript have the following competing interests: [insert competing interests here]*<br><br>* typeset	
**Ethics Statement**<br><br>Enter an ethics statement for this submission. This statement is required if the study involved:<br><br>• Human participants<br>• Human specimens or tissue<br>• Vertebrate animals or cephalopods<br>• Vertebrate embryos or tissues<br>• Field research<br><br>Write "N/A" if the submission does not require an ethics statement.<br><br>General guidance is provided below. Consult the submission guidelines for detailed instructions. **Make sure that all information entered here is included in the**	N/A

**Methods section of the manuscript.**<br><br>**Format for specific study types**<br><br>**Human Subject Research (involving human participants and/or tissue)**<br>• Give the name of the institutional review board or ethics committee that approved the study<br>• Include the approval number and/or a statement indicating approval of this research<br>• Indicate the form of consent obtained (written/oral) or the reason that consent was not obtained (e.g. the data were analyzed anonymously)<br><br>**Animal Research (involving vertebrate animals, embryos or tissues)**<br>• Provide the name of the Institutional Animal Care and Use Committee (IACUC) or other relevant ethics board that reviewed the study protocol, and indicate whether they approved this research or granted a formal waiver of ethical approval<br>• Include an approval number if one was obtained<br>• If the study involved *non-human primates*, add *additional details* about animal welfare and steps taken to ameliorate suffering<br>• If anesthesia, euthanasia, or any kind of animal sacrifice is part of the study, include briefly which substances and/or methods were applied<br><br>**Field Research**<br><br>Include the following details if this study involves the collection of plant, animal, or other materials from a natural setting:<br>• Field permit number<br>• Name of the institution or relevant body that granted permission	
**Data Availability**<br><br>Authors are required to make all data underlying the findings described fully available, without restriction, and from the time of publication. PLOS allows rare exceptions to address legal and ethical concerns. See the PLOS Data Policy and FAQ for detailed information.	No - some restrictions will apply

A Data Availability Statement describing where the data can be found is required at submission. Your answers to this question constitute the Data Availability Statement and **will be published in the article**, if accepted.<br><br>**Important:** Stating 'data available on request from the author' is not sufficient. If your data are only available upon request, select 'No' for the first question and explain your exceptional situation in the text box.<br><br>Do the authors confirm that all data underlying the findings described in their manuscript are fully available without restriction?	
**Describe where the data may be found in full sentences. If you are copying our sample text, replace any instances of XXX with the appropriate details.**<br><br>• If the data are **held or will be held in a public repository**, include URLs, accession numbers or DOIs. If this information will only be available after acceptance, indicate this by ticking the box below. For example: *All XXX files are available from the XXX database (accession number(s) XXX, XXX.*).<br>• If the data are all contained **within the manuscript and/or Supporting Information files**, enter the following: *All relevant data are within the manuscript and its Supporting Information files.*<br>• If neither of these applies but you are able to provide **details of access elsewhere**, with or without limitations, please do so. For example:<br><br>*Data cannot be shared publicly because of [XXX]. Data are available from the XXX Institutional Data Access / Ethics Committee (contact via XXX) for researchers who meet the criteria for access to confidential data.*<br><br>*The data underlying the results*	https://dataverse.harvard.edu/dataset.xhtml?persistentId=doi:10.7910/DVN/RRXQNT

presented in the study are available from (include the name of the third party and contact information or URL).<br>• This text is appropriate if the data are owned by a third party and authors do not have permission to share the data.<br><br>* typeset	
Additional data availability information:	

Manuscript Click here to access/download;Manuscript;Manuscript.docx

Associated factors and socio-economic inequality in the prevalence of thinness and stunting among adolescent boys and girls in Uttar Pradesh and Bihar, India

Pradeep Kumar[1], Shobhit Srivastava[1], Shekhar Chauhan[2], Ratna Patel[3], Strong Pillar Marbaniang[1*], Preeti Dhillon[4]

[1] Ph.D. Research Scholar, Department of Mathematical Demography & Statistics, International Institute for Population Sciences, Mumbai, India

[2] Ph.D. Research Scholar, Department of Population Policies and Programmes, International Institute for Population Sciences, Mumbai, India

[3] Ph.D. Research Scholar, Department of Public Health and Mortality Studies, International Institute for Population Sciences, Mumbai, India

[4] Assistant Professor, Department of Mathematical Demography & Statistics, International Institute for Population Sciences, Mumbai, India

*** Corresponding author:** marbaniangstrong@gmail.com

Pradeep Kumar: pradeepmpsiips@gmail.com

Shobhit Srivastava: shobhitsrivastava889@gmail.com

Shekhar Chauhan: shekhariips2486@gmail.com

Ratna Patel: ratnapatelbhu@gmail.com

Strong Pillar Marbaniang: marbaniangstrong@gmail.com

Preeti Dhillon: pdhillon.iips@gmail.com

Associated factors and socio-economic inequality in the prevalence of thinness and stunting among adolescent boys and girls in Uttar Pradesh and Bihar, India

Abstract

Background: Despite economic growth observed in developing countries, under-nutrition still continues to be a major health problem. Undernutrition in adolescence can disrupt normal growth and puberty development and may have long-term impact. Therefore, it is important to study the undernutrition among adolescents. This study aimed to assess the prevalence and the associated factors of stunting, thinness and the coexistence of both (stunting and thinness) among the adolescent belonging to Uttar Pradesh and Bihar, India.

Methods: The study utilized data from Understanding the Lives of Adolescents and Young Adults (UDAYA) project survey, which was conducted in two Indian states Uttar Pradesh and Bihar, in 2016 by Population Council under the guidance of Ministry of Health and Family Welfare, Government of India. Utilizing information on 20,594 adolescents aged 10-19 years (adolescent boys-5,969 and adolescent girls-14,625), the study examined three outcome variables, i.e., thinness, stunting, and co-existence of both. The study used descriptive and bivariate analysis. Furthermore, the study examined income-related inequality in stunting and thinness through concentration index. At last, the study used Wagstaff decomposition analysis to decompose the concentration index.

Results: The prevalence of thinness was higher among adolescent boys as compared to girls (25.8% vs. 13.1%). However, stunting was more prevalent among girls (25.6%) than in boys (39.3%). The odds of stunting and thinness were higher among late adolescents, uneducated adolescents, adolescents not working, and poorest adolescents. Media exposure and wealth index contributed significantly to the inequality in the prevalence of thinness among adolescents, whereas, wealth index was the only significant contributor to the inequality in the prevalence of stunting among adolescents.

Conclusion: The study provides an understanding that stunting and thinness is a significant public health concern among adolescents, and there is a need to tackle the issue comprehensively. By tackling the issue comprehensively, we mean that the state government of Uttar Pradesh and Bihar shall screen, assess, and monitor the nutritional status of adolescent boys and girls. The interventions shall focus towards both boys as well as girl adolescents, and

particular emphasis should be given to adolescents who belonged to poor households. Also, efforts should be taken by stakeholders to increase family wealth status.

Keywords: Stunting; Thinness; Adolescents; Socio-economic inequality; Decomposition analysis.

Associated factors and socio-economic inequality in the prevalence of thinness and stunting among adolescent boys and girls in Uttar Pradesh and Bihar, India

Introduction

Adolescence is a part of life between childhood and adulthood, from ages 10 to 19 years. [1] It is usually divided into early adolescence (10-14 years) and late adolescence (15-19 years) [1]. It is a crucial stage of human development and an important time for starting the foundation of good health. Today adolescent population constitute about 1.2 billion of the world population (7.2 billion), and nearly 350 million live in South Asia the highest than any other region [2]. In absolute number, India has 253 million adolescent which constitute about 20.9% of the total population [3]. Adolescence is a period of physical growth, cognitive transformation, and reproductive maturation in the life cycle which lead to the high requirements of macro or micronutrients or both [4]. During this period, up to 45% of skeletal growth takes place, and 15% to 25% of adult height is achieved [5]. Nutrition influence growth and development throughout infancy, childhood and adolescent; however, the requirements of nutrient are high during the adolescent period than any other period after birth [6]. Furthermore, with increasing age, adolescent's personal choices and preferences become a priority over eating habits acquired in the family, and they have gradually more control over what they eat, when and where [5].

Macro and micronutrients deficiency in the body are due to inadequate food intake, low nutrient content of the food and frequent infections. For measuring the nutritional status of adolescent, the World Health Organisation (WHO) has recommended the use of low height-for-age (stunting) and low BMI-for-age (thinness) [7], with the former indicating chronic undernutrition and the later indicating acute undernutrition [8,9]. Despite economic growth observed in developing countries, under-nutrition still continues to be a major health problem [10]. Recent estimates showed that the prevalence of thinness among Indian adolescent was

26.7% [11], and the prevalence of stunting was 34.1% [1]. Studies from two Indian eastern states (Chhattisgarh and Odisha) shows that the prevalence of thinness among adolescent girls aged 10-14 and 15-19 years was 17.1% and 9.6% respectively [12]. Another study among 5521 adolescent in rural West Bengal shows that the prevalence of stunting was 23.3% among males and 26.9% among females [3]. Existing evidence acknowledge that socioeconomic status, age, family size, parents education status, lack of latrine, and poor water supply were the commonly mentioned factors that influence the nutritional status of the adolescent [4,13].

Undernutrition in adolescence can disrupt normal growth and puberty development [6] and increased the risk to infectious diseases [14]. It is also associated with adult lower educational achievement and income status [15]. Undernourished adolescent girls that enter pregnancy are more likely to give birth to a baby of low birth weight or intrauterine growth restricted baby that is more susceptible to metabolic disorder later in life [13]. Nutrition is also vital in reproduction, which includes the safe delivery of infants. MÖLler and Lindmark, (1997) showed that the failure to undergo natural delivery was associated with the height of the mother, which is influence by nutritional status during childhood and adolescent [16].

Given the above background, the present study aimed to assess the prevalence and the associated factors of stunting, thinness and the coexistence of both among the adolescent belonging to Uttar Pradesh and Bihar. Given that the adolescent represents the next generation, limited studies have been done to understand the determinants and the inequality of adolescent undernutrition [9,17,18]. The findings will help to provide important evidence for future planning and implementation of nutritional policies and programmes which aimed to improve the nutritional status of adolescents.

Methods:

Data:

The data for this study was carried out from Understanding the Lives of Adolescents and Young Adults (UDAYA) project survey, which was conducted in two Indian states Uttar Pradesh and Bihar, in 2016 by Population Council under the guidance of Ministry of Health and Family Welfare, Government of India. With the written consent of respondents, the survey gathered information on family, media, community environment, assets acquired in adolescence, and quality of transitions to young adulthood indicators. With the use of a multi-stage systematic sampling design, the survey provides the estimates for states as a whole as well as urban and rural areas of the states. The detailed sampling design, data collection procedure, and survey

tools available elsewhere [19]. The sample size for Uttar Pradesh and Bihar was 10,350 and 10,350 adolescents aged 10-19 years, respectively. The required sample for each sub-group of adolescents was determined at 920 younger boys, 2,350 older boys, 630 younger girls, 3,750 older girls, and 2,700 married girls in both states. The effective sample size for this study was 20,594 adolescents aged 10-19 years (adolescent boys-5,969 and adolescent girls-14,625) [19].

This study uses data which is secondary in nature and therefore does not require any ethical approval from any institutional review board. The data collection for UDAYA survey was approved by Population Council, New Delhi and ethical review board of Population Council, New Delhi approved the questionnaire that was used in the field work. The written consent from the participants was taken before undertaking the study.

Variable description

Outcome variables

There were three outcome variables, i.e., thinness, stunting, and co-existence of both. Thinness among adolescents was constructed using WHO-recommended cut-off points [20]. BMI-for-age Z-score < -2SD was cut-off for thinness. The other variable, i.e., stunting, was constructed using the WHO recommended cut-offs [21]. Height -for-age Z-score of <= -2SD was cut-for stunting among adolescents. The co-existence of thinness and stunting was generated using egen command in STATA 14. It was defined if thinness and stunting co-exist in a single adolescent. The analysis was bifurcated for adolescent boys and adolescent girls because the data only give estimates separately for adolescent boys and girls.

Explanatory variables

1. Age was grouped into two categories i.e., early adolescents (10-14 years) and late adolescents (15-19 years).
2. Education was recorded as no schooling, 1-7, 8-9, and 10 and above years of education.
3. Working status was recoded as not working "no" and working "yes."
4. Media exposure was coded as no exposure, rare exposure, and frequent exposure.
5. Wealth index was recoded as poorest, poorer, middle, richer, and richest.
6. Caste was recoded as Scheduled caste and Scheduled tribe (SC/ST) and non-SC/ST.

7. Religion was recoded as Hindu and non-Hindu. The category of non-Hindu was recoded as so because the frequency of other religions was very low; therefore, analytical purpose the recoding was done in a respective manner.
8. The residence was available in data as urban and rural.
9. Data were available for two states i.e., Uttar Pradesh and Bihar, as the survey was conducted in these two states only.

Statistical analysis

Descriptive and bivariate analysis was done to understand the sample distribution of the study population and get the estimates of thinness, stunting, and co-existence of both separately for adolescent boys and girls. Further, logistic regression analysis was used to identify the factors associated with thinness, stunting, and co-existence of both. Lastly, regression-based decomposition analysis was used to find the absolute contribution of the factors for outcome variables.

Concentration Index (CI)

Income-related inequality in infant mortality was quantified by the concentration index (CI) and the concentration curve (CC), using the wealth score as the socio-economic indicator and binary outcome as thinness, stunting, and co-existence of both. The concentration curve is obtained by plotting the cumulative proportion of poor health against the cumulative proportion of the population ranked by the socio-economic indicator. The concentration index can be written as follows:

$$\boldsymbol{C} = \frac{\boldsymbol{2}}{\boldsymbol{\mu}}\boldsymbol{cov}(\boldsymbol{y}_{\boldsymbol{i},}\boldsymbol{R}_{\boldsymbol{i}})$$

Where, C is the concentration index; y_i is the outcome variable index; $\boldsymbol{R}$ is the fractional rank of individual $\boldsymbol{i}$ in the distribution of socio-economic position; $\boldsymbol{\mu}$ is the mean of the outcome variable of the sample and $\boldsymbol{cov}$ denotes the covariance [22].

The index value lies between -1 to +1. If the curve lies above the line of equality, the concentration index takes a negative value, indicating a disproportionate concentration of inequality among the poor (pro-rich). Conversely, if the curve lies below the line of equality, the concentration index takes a positive value, indicating a disproportional concentration of inequality among the rich (pro-poor). In the absence of socio-economic related inequality, the concentration index is zero.

Decomposition of the concentration index

The study used Wagstaff decomposition analysis to decompose the concentration index. Wagstaff's decomposition demonstrated that the concentration index could be decomposed into the contributions of each factor to the income-related inequalities [23]. Based on the linear regression relationship between the outcome variabley_i, the intercept α, the relative contribution of x_{ki} and the residual error ε_i

$$y_i = \alpha + \sum \beta_k x_{ki} + \varepsilon_i$$

Where ε_i is an error term, given the relationship between y_i andx_{ki}, the CI for y (C) can be rewritten as:

$$C = \sum \left(\frac{\beta_k \bar{x}_k}{\mu}\right) C_k + \frac{GC\varepsilon}{\mu} / \mu$$

Where μ is the mean of y_i, $\bar{x}_k$, is the mean of x_k, β_k is the coefficient from a linear regression of outcome variable, $C_k$is the concentration index for x_k (defined analogously to C, and GC_ε is the generalized concentration index for the error term (ε_i).

Here C is the outcome of two components: First, the determinants or 'explained' factors. The explained factors indicate that the proportion of inequalities in the outcome (thinness, stunting, and co-existence of both) variable is explained by the selected various socio-economic groups, i.e., x_k. Second, a residual or 'unexplained' factor $\left(\frac{GC\varepsilon}{\mu} / \mu\right)$, indicating the inequality in health variables that cannot be explained by selected explanatory factors across various socio-economic groups.

Results:

Figure 1 display the prevalence of thinness, stunting, and co-existing of both among adolescents aged 10-19 years. The prevalence of thinness (25.8% vs. 13.1%) was higher among adolescent boys as compared to girl counterparts. However, the stunting was more prevalent among girls (25.6%) than in boys (39.3%). Nearly 10 per cent of adolescent boys and six per cent of adolescent girls suffered from co-existence of both indicators.

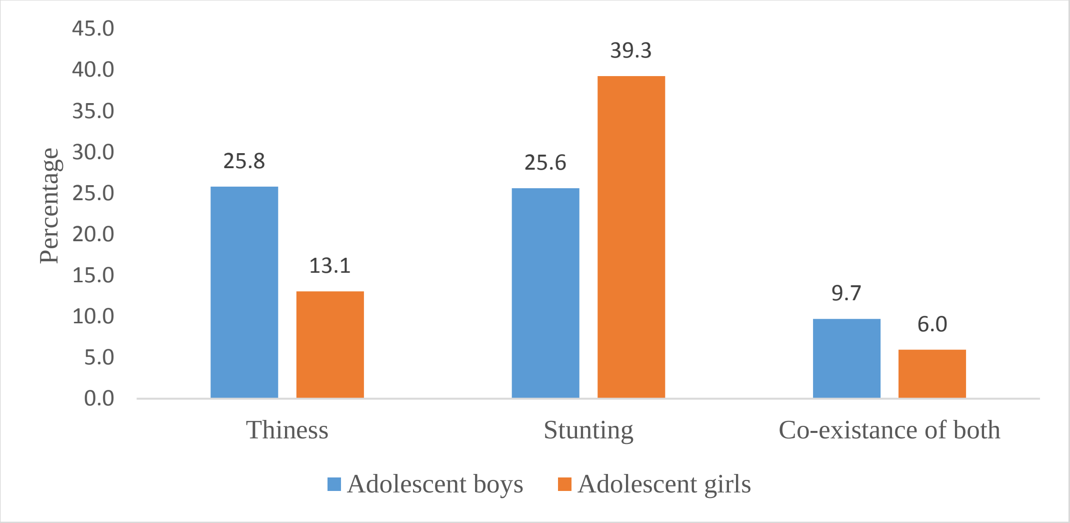

Figure 1: Thinness, stunting, and co-existence of both among adolescents aged 10-19 years

The socio-demographic profile of the study population was presented in **Table 1**. A higher proportion of the study population was from the late adolescents' group (boys-65% and girls-88.7%), and about one-fourth of boy and one-third of adolescent girls had 10 & above years of schooling. Nearly 27 per cent of adolescent boys and 17 per cent adolescent girls were working, and three-fourth of boys and about half of the adolescent girls used mass media frequently.

Table-1 Socio-demographic profile of adolescents aged 10-19 years

Background characteristics	Adolescent boys		Adolescent girls	
	Sample	Percentage	Sample	Percentage
**Age (years)**				
Early adolescents (10-14)	2,084	34.9	1,653	11.3
Late adolescents (15-19)	3,885	65.1	12,972	88.7
**Educational status (years)**				
No schooling	190	3.2	1,890	12.9
1-7	2,497	41.8	3,939	26.9
8-9	1,754	29.4	4,093	28.0
10 and above	1,528	25.6	4,703	32.2
**Working status**				
No	4,377	73.3	12,179	83.3
Yes	1,592	26.7	2,446	16.7
**Media exposure**				
No exposure	335	5.6	2,703	18.5
Rare	1,078	18.1	4,212	28.8
Frequent	4,555	76.3	7,710	52.7
**Wealth Index**				
Poorest	704	11.8	1,971	13.5
Poorer	1,193	20.0	2,735	18.7
Middle	1,374	23.0	3,188	21.8
Richer	1,391	23.3	3,577	24.5
Richest	1,308	21.9	3,154	21.6

**Caste**					
	SC/ST	1,605	26.9	3,784	25.9
	Non-SC/ST	4,364	73.1	10,841	74.1
**Religion**					
	Hindu	5,024	84.2	11,540	78.9
	Non-Hindu	945	15.8	3,085	21.1
**Residence**					
	Urban	1,030	17.3	2,356	16.1
	Rural	4,939	82.7	12,269	83.9
**States**					
	Uttar Pradesh	4,069	68.2	9,855	67.4
	Bihar	1,900	31.8	4,770	32.6
**Total**		5,969	100.0	14,625	100.0

SC/ST: Scheduled Caste/Scheduled Tribe

Table 2 shows the percentage distribution of thinness, stunting, and co-existence of both among adolescents aged 10-19 years by various background characteristics. The prevalence of thinness was higher among early adolescents groups, irrespective of their gender. Moreover, stunting was more prevalent among late adolescents (boy-27.3% and girl-42.9%). Nearly 11 per cent of boys and 8% of early adolescent girls suffered from co-existence of both. The prevalence of thinness (30.4%) and co-existence of both (13%) were highest among adolescent boys who had 1-7 years of schooling, and it was least among those who had 10 & above years of education. In the case of adolescent girls, a negative association was found between years of schooling and co-existence of thinness and stunting. Thinness was higher among adolescents who were not working (boy-27.6% and girl-13.5%), and this finding was revered for stunting. The prevalence of thinness (boy-23.2% and girl-12.1%), stunting (boy-23.9% and girl-36.8%), and co-existence of both (boy-8% and girl-4.8%) were lowest among adolescents who used mass media frequently.

On the other hand, thinness, stunting, and co-existence of both have a negative relationship with wealth index for adolescent boys. For instance, these indicators was highest among poorest adolescents (thinness-33.9%, stunting-35% and co-existence-16.5%) and lowest in richest ones (thinness-16.5%, stunting-14.4% and co-existence-4%). In the case of adolescent girls, a similar relationship was found for the co-existence of both indicators. The prevalence of thinness (boy-31% and girl-13.7%), stunting (boy-33.4% and girl-45.6%), and co-existence of both (boy-11.8% and girl-8%) were highest among SC/ST adolescents compared to non-SC/ST ones, irrespective to their gender. Similarly, rural adolescents suffered more from thinness, stunting, and co-existence of both than urban counterparts, irrespective of their gender.

Table-2 Percentage distribution for thinness, stunting and co-existence of both by background characteristics among adolescents aged 10-19 years

Background characteristics	Adolescent boys			Adolescent girls		
	Thinness (N=3242)	Stunting (N=3190)	Both (N=3185)	Thinness (N=4297)	Stunting (N=4396)	Both (N=3979)
**Age (years)**						
Early adolescents (10-14)	29.3	24.5	11.3	19.0	32.3	7.6
Late adolescents (15-19)	20.7	27.3	7.3	9.7	42.9	5.0
**Educational status (years)**						
No schooling	23.2	29.0	6.8	12.7	49.6	7.4
1-7	30.4	28.1	13.0	16.7	38.5	7.1
8-9	21.9	24.3	6.9	9.6	38.2	5.3
10 and above	14.8	17.3	1.9	10.3	36.0	3.9
**Working status**						
No	27.6	24.5	10.6	13.5	38.9	6.0
Yes	19.3	30.0	6.6	10.1	42.2	6.0
**Media exposure**						
No exposure	32.2	28.3	11.3	14.9	41.4	8.5
Rare	33.7	31.4	15.7	13.6	42.3	6.5
Frequent	23.2	23.9	8.0	12.1	36.8	4.8
**Wealth Index**						
Poorest	33.9	35.2	16.5	17.2	44.9	9.3
Poorer	32.3	30.3	12.0	11.7	45.3	6.5
Middle	29.1	29.5	12.1	13.3	42.1	7.7
Richer	20.6	22.6	6.5	12.2	37.9	4.3
Richest	16.5	14.4	4.0	12.0	28.2	3.3
**Caste**						
SC/ST	31.0	33.4	11.8	13.7	45.6	8.0
Non-SC/ST	23.9	22.8	9.0	12.8	36.8	5.2
**Religion**						
Hindu	25.9	26.2	10.1	12.3	40.1	5.9
Non-Hindu	25.6	22.5	7.9	16.1	36.1	6.1
**Residence**						
Urban	20.4	19.0	5.5	12.5	36.0	4.2
Rural	26.9	27.0	10.6	13.2	39.8	6.3
**States**						
Uttar Pradesh	25.5	27.3	10.0	14.1	38.0	6.1
Bihar	26.4	22.3	9.1	11.1	42.1	5.6

SC/ST: Scheduled Caste/Scheduled Tribe

Estimates from logistic regression analysis for thinness, stunting, and co-existence of both among adolescents aged 10-19 years were presented in **Table 3**. The likelihood of stunting [OR: 1.79; CI: 1.39-2.3] was 79% significantly more likely among late adolescent boys than early ones. In case of adolescent girls, the likelihood of thinness was 52% less likely [OR: 0.48; CI: 0.37-0.62], stunting was 2.25 times more likely [OR: 2.25; CI: 1.9-2.67] and co-existence of both was 38% less likely [OR: 0.62; CI: 0.43-0.89] among late adolescents compared to early adolescents. The odds of thinness [OR: 0.50; CI: 0.28-0.87], stunting [OR: 0.34; CI: 0.2-0.6], and co-existence of both [OR: 0.18; CI: 0.07-0.48] was 50%, 66% and 82% significantly less likely among adolescent boys who had 10 & above years of schooling respectively than those had no schooling. Moreover, the likelihood of thinness [OR: 0.60; CI: 0.41-0.87],

stunting [OR: 0.65; CI: 0.52-0.81], and co-existence of both [OR: 0.54; CI: 0.32-0.9] was 40%, 35% and 46% significantly less likely among adolescent girls who had 8-9 years of schooling respectively compared to those who had no schooling. The likelihood of thinness [OR: 0.73; CI: 0.57-0.93] and co-existence of both (thinness and stunting) [OR: 0.58; CI: 0.38-0.89] was 27% and 32% significantly less likely among working adolescent boys respectively than not working counterparts. With reference to poorest category, adolescent boys who were belonged to wealthier families, 41%, 61%, and 68% less likely to suffer from thinness [OR: 0.59; CI: 0.43-0.82], stunting [OR: 0.39; CI: 0.28-0.56] and co-existence of both [OR: 0.32; CI: 0.19-0.55] respectively. Similarly, stunting [OR: 0.56; CI: 0.43-0.72], and co-existence of both (thinness and stunting) [OR: 0.54; CI: 0.30-0.98] were 44% and 46% significantly less likely among adolescent girls who belonged to richest wealth quintile. Moreover, non-SC/ST adolescent boys were 20% and 23% significantly less likely to suffer from thinness [OR: 0.80; CI: 0.65-0.97] and stunting [OR: 0.77; CI: 0.63-0.95] respectively compared to SC/ST counterparts. The odds of stunting were 24% significantly lower among adolescent girls [OR: 0.76; CI: 0.65-0.88] who belonged to the non-SC/ST community than SC/ST ones.

Table-3 Logistic regression estimates for thinness, stunting and co-existence of both by background characteristics among adolescents aged 10-19 years

Background characteristics	Adolescent boys			Adolescent girls		
	**Thinness (N=3242)**	**Stunting (N=3190)**	**Both (N=3185)**	**Thinness (N=4297)**	**Stunting (N=4396)**	**Both (N=3979)**
	**OR (95% CI)**	**OR (95% CI)**	**OR (95% CI)**	**OR (95% CI)**	**OR (95% CI)**	**OR (95% CI)**
**Age (years)**						
Early adolescents (10-14)	Ref.	Ref.	Ref.	Ref.	Ref.	Ref.
Late adolescents (15-19)	1.14(0.89,1.45)	1.79*(1.39,2.3)	1.35(0.91,1.99)	0.48*(0.37,0.62)	2.25*(1.9,2.67)	0.62*(0.43,0.89)
**Educational status (years)**						
No schooling	Ref.	Ref.	Ref.	Ref.	Ref.	Ref.
1-7	0.96(0.58,1.59)	0.73(0.45,1.21)	0.88(0.42,1.87)	0.89(0.63,1.25)	0.85(0.68,1.05)	0.80(0.5,1.27)
8-9	0.72(0.42,1.22)	0.49*(0.29,0.83)	0.48(0.22,1.08)	0.60*(0.41,0.87)	0.65*(0.52,0.81)	0.54*(0.32,0.9)
10 and above	0.50*(0.28,0.87)	0.34*(0.2,0.6)	0.18*(0.07,0.48)	0.98(0.67,1.43)	0.55*(0.44,0.7)	0.62(0.35,1.1)
**Working status**						
No	Ref.	Ref.	Ref.	Ref.	Ref.	Ref.
Yes	0.73*(0.57,0.93)	1.05(0.83,1.34)	0.58*(0.38,0.89)	0.80(0.58,1.1)	1.12(0.92,1.36)	0.96(0.62,1.5)
**Media exposure**						
No exposure	Ref.	Ref.	Ref.	Ref.	Ref.	Ref.
Rare	1.25(0.82,1.9)	1.15(0.75,1.77)	1.42(0.77,2.59)	1.11(0.82,1.51)	1.12(0.92,1.37)	0.91(0.59,1.39)
Frequent	1.01(0.68,1.5)	1.01(0.67,1.51)	0.99(0.55,1.78)	0.95(0.69,1.3)	1.02(0.83,1.25)	0.81(0.52,1.26)
**Wealth Index**						
Poorest	Ref.	Ref.	Ref.	Ref.	Ref.	Ref.
Poorer	0.82(0.61,1.11)	0.79(0.58,1.07)	0.7(0.46,1.07)	0.83(0.59,1.16)	0.95(0.76,1.18)	0.85(0.53,1.38)
Middle	0.88(0.66,1.18)	0.79(0.59,1.06)	0.72(0.48,1.08)	0.96(0.69,1.34)	0.89(0.72,1.11)	1.18(0.75,1.86)
Richer	0.80(0.59,1.09)	0.58*(0.42,0.8)	0.55*(0.35,0.87)	0.89(0.63,1.25)	0.78*(0.62,0.98)	0.72(0.43,1.19)
Richest	0.59*(0.43,0.82)	0.39*(0.28,0.56)	0.32*(0.19,0.55)	0.79(0.54,1.15)	0.56*(0.43,0.72)	0.54*(0.30,0.98)
**Caste**						
SC/ST	Ref.	Ref.	Ref.	Ref.	Ref.	Ref.
Non-SC/ST	0.80*(0.65,0.97)	0.77*(0.63,0.95)	0.90(0.66,1.22)	0.89(0.70,1.12)	0.76*(0.65,0.88)	0.74(0.53,1.05)
**Religion**						
Hindu	Ref.	Ref.	Ref.	Ref.	Ref.	Ref.
Non-Hindu	1.07(0.85,1.35)	0.98(0.76,1.27)	0.87(0.58,1.3)	1.31*(1.03,1.66)	0.93(0.78,1.1)	1.35(0.94,1.93)
**Residence**						
Urban	Ref.	Ref.	Ref.	Ref.	Ref.	Ref.
Rural	1.13(0.93,1.36)	1.15(0.94,1.41)	1.31(0.95,1.81)	1.0(0.81,1.25)	0.97(0.84,1.12)	0.94(0.68,1.31)
**States**						
Uttar Pradesh	Ref.	Ref.	Ref.	Ref.	Ref.	Ref.
Bihar	0.82*(0.69,0.97)	0.66*(0.55,0.79)	0.61*(0.46,0.8)	0.74*(0.61,0.9)	0.97(0.85,1.1)	0.71*(0.52,0.95)

Figure 2a, 2b, and 2c presents the concentration curve for thinness, stunting and co-existence of both among adolescent boys and girls aged 10-19 years. If the curve is formed below the line of equality than the inequality is concentrated towards rich and vice-versa. Moreover, more the area between line of equality and curve higher the inequality. Uttar Pradesh and Bihar witnessed a CI value of -0.15 for adolescent boys and -0.04 for adolescent girls which depicts pro-rich bias of thinness among adolescents (**figure 2a**). Moreover, highest inequality of stunting was witnessed among boys (-0.16) than girls (-0.09) adolescent (**figure 2b**). Additional, for the co-existence of both thinness and stunting, the inequality was increased drastically among boys and girls adolescent. For instance, the highest inequality of co-existence of both thinness and stunting was observed among boys (-0.25) compared to girls (-0.17) adolescent (**figure 2c**). In other words, thinness, stunting and co-existence of both (thinness and stunting) was concentrated among poor adolescents only and it was highest among adolescent boys.

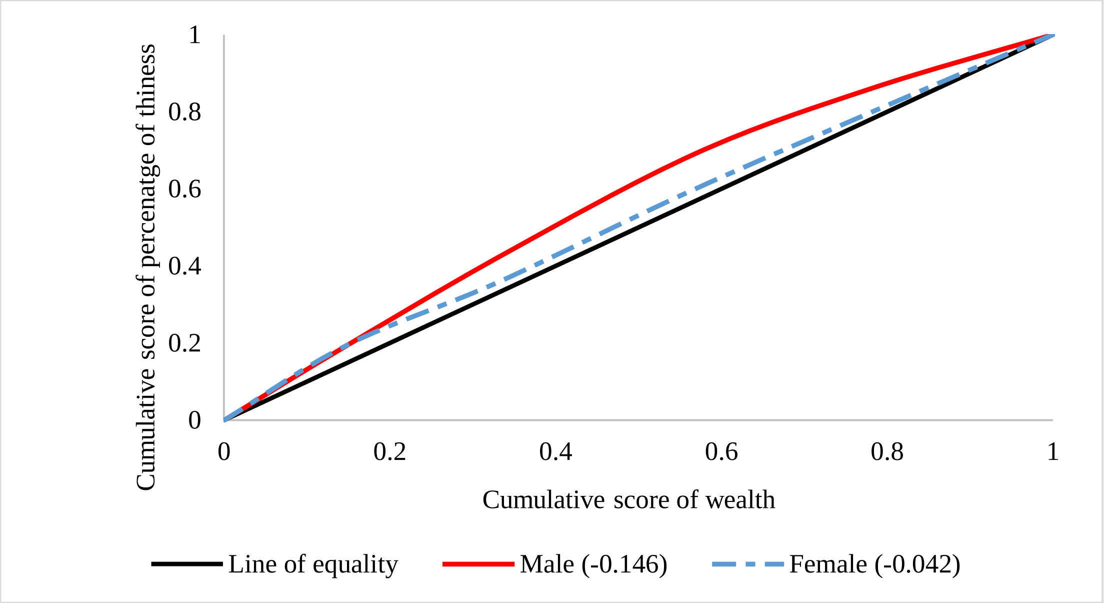

Figure 2a Concentration curve for thinness among adolescents aged 10-19 years

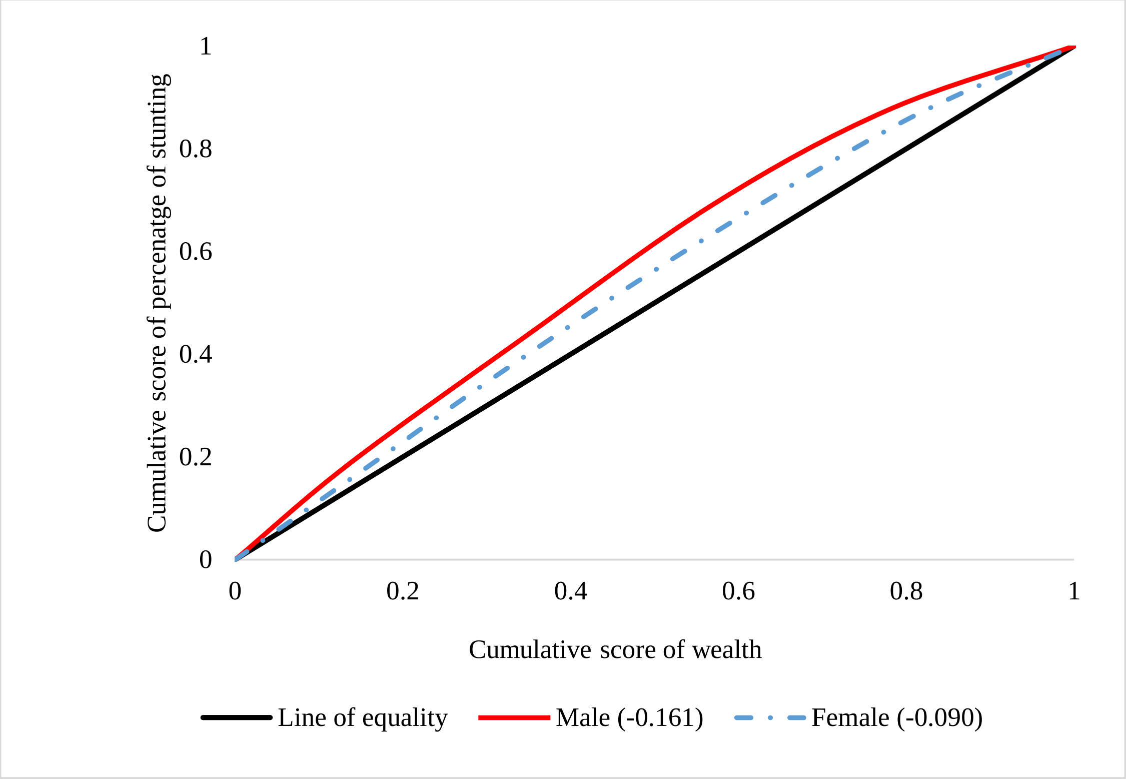

Figure 2b Concentration curve for stunting among adolescents aged 10-19 years

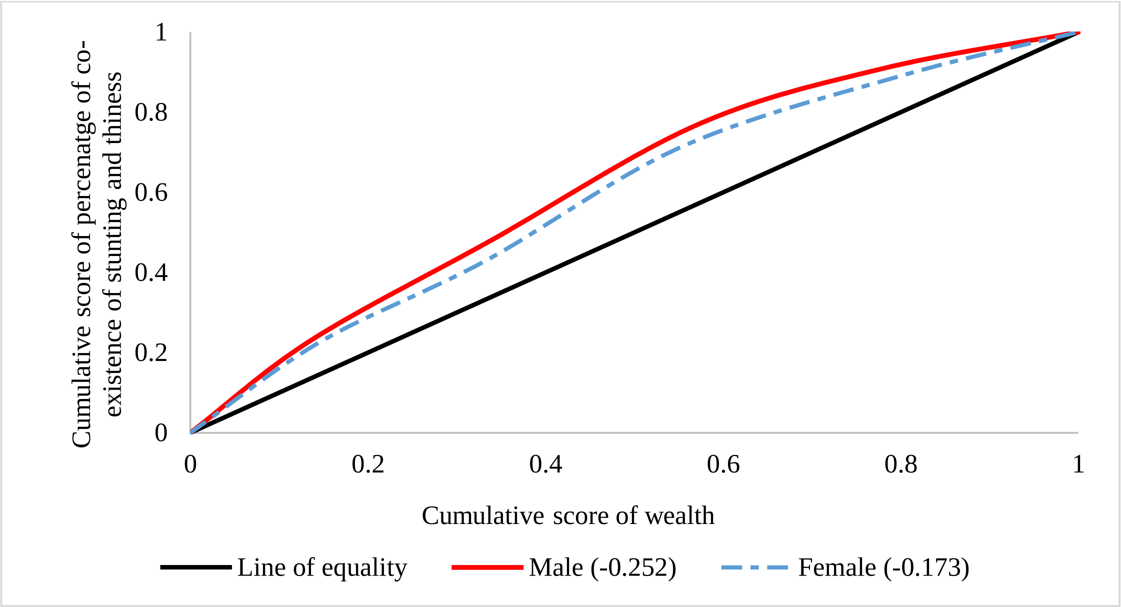

Figure 2c Concentration curve for co-existence of stunting and thinness among adolescents aged 10-19 years

Table-4a, 4b and 4c represent estimates of decomposition analysis for the contribution of various explanatory variables for thinness, stunting, and co-existence of thinness and stunting among adolescents. The percentage contribution is the column to be interpreted for depicting the percentage contribution of factors for explaining socio-economic status (SES) related inequality thinness, stunting, and co-existence of thinness and stunting among adolescents. The negative and positive signs are to make the total contribution as 100 and dependents upon the sign elasticity and concentration index (CI) of the table. The absolute contribution is the product of elasticity and CI. Moreover, the individual contribution is the division of the absolute contribution of the individual factors and total absolute contribution. Hence the magnitude of the percentage contribution depends on the elasticity and CI.

Wealth status contributed about 80 per cent to explain SES related inequality followed by educational status (15 per cent) and media exposure (12 per cent) for thinness among adolescent boys. Whereas among adolescent girls, wealth status contributed about 66 per cent of SES related inequality followed by media exposure (58 per cent) and age (16 per cent) for thinness.

For stunting among adolescents boys, wealth status contributed 85 per cent of SES related inequality followed by educational status (24 per cent) and caste (10 per cent). In the case of adolescent girls, factors were the same as in adolescent boys i.e.; wealth status contributed 84 per cent followed by educational status (24 per cent) and caste (9 per cent) to explain SES related inequality for stunting.

For co-existence of thinness and stunting among adolescent boys, wealth status contributed 79 per cent to explain SES related inequality followed by educational status (26 per cent) and media exposure (12 per cent). Whereas in case of co-existence of thinness and stunting among adolescent girls, wealth status contributed 71 per cent to explain SES related inequality followed by media exposure (19 per cent) and caste (13 per cent).

Table-4a Estimates of decomposition analysis for contribution of various explanatory variables for thinness among adolescents aged 10-19 years

Background characteristics	Adolescent boys					Adolescent girls				
	**Elasticity**	**CI**	**Absolute contribution**	**% contribution**	**Tot al**	**Elasticity**	**CI**	**Absolute contribution**	**% contribution**	**Total**
**Age (years)**										
Early adolescents (10-14)										
Late adolescents (15-19)	0.006	0.087	0.001	-1.3	-1.3	-0.060	0.016	-0.001	16.1	16.1
**Educational status (years)**										
No schooling										
1-7.	0.032	-0.092	-0.003	8.1		-0.002	-0.088	0.000	-3.2	
8-9.	-0.001	0.064	0.000	0.3		-0.007	0.031	0.000	3.2	
10 and above	-0.009	0.268	-0.002	6.5	14.8	0.001	0.277	0.000	-4.8	-4.8
**Working status**										
No										
Yes	-0.022	-0.152	0.003	-9.1	-9.1	-0.004	-0.255	0.001	-17.7	-17.7
**Media exposure**										
No exposure										
Rare	0.006	-0.304	-0.002	5.1		-0.003	-0.226	0.001	-9.7	
Frequent	-0.021	0.120	-0.003	6.7	11.8	-0.016	0.261	-0.004	67.7	58.1
**Wealth Index**										
Poorest										
Poorer	0.000	-0.526	0.000	-0.5		-0.010	-0.518	0.005	-82.3	
Middle	-0.006	-0.091	0.001	-1.3		-0.006	-0.107	0.001	-11.3	
Richer	-0.021	0.346	-0.007	19.6		-0.010	0.367	-0.004	59.7	
Richest	-0.030	0.778	-0.023	62.4	80.1	-0.008	0.808	-0.006	100.0	66.2
**Caste**										
SC/ST										
Non-SC/ST	-0.043	0.089	-0.004	10.2	10.2	-0.012	0.086	-0.001	16.1	16.1
**Religion**										
Hindu										
Non-Hindu	0.004	0.176	0.001	-1.9	-1.9	0.007	0.102	0.001	-11.3	-11.3
**Residence**										
Urban										
Rural	-0.002	-0.091	0.000	-0.3	-0.3	0.008	-0.067	-0.001	9.7	9.7
**States**										
Uttar Pradesh										

Bihar	-0.010	-0.174	0.002	-4.3	-4.3	-0.011	-0.176	0.002	-32.3	-32.3
**Calculated CI**			-0.037	100.0				-0.006	100.0	
**Actual CI**			-0.146					-0.042		
**Residual**			-0.109					-0.035		

CI: Concentration Index; SC/ST: Scheduled Caste/Scheduled Tribe; %: Percentage

Table-4b Estimates of decomposition analysis for contribution of various explanatory variables for stunting among adolescents aged 10-19 years

**Background characteristics**	**Adolescent boys**					**Adolescent girls**				
	**Elasticity**	**CI**	**Absolute contribution**	**% contribution**		**Elasticity**	**CI**	**Absolute contribution**	**% contribution**	
**Age (years)**										
Early adolescents (10-14)										
Late adolescents (15-19)	0.055	0.088	0.005	-12.5	-12.5	0.103	0.016	0.002	-4.9	-4.9
**Educational status (years)**										
No schooling										
1-7.	0.028	-0.091	-0.003	6.8		-0.011	-0.092	0.001	-3.0	
8-9.	-0.010	0.064	-0.001	1.6		-0.023	0.036	-0.001	2.5	
10 and above	-0.022	0.273	-0.006	15.3	23.6	-0.029	0.272	-0.008	24.0	23.5
**Working status**										
No										
Yes	-0.004	-0.156	0.001	-1.6	-1.6	-0.002	-0.260	0.001	-1.8	-1.8
**Media exposure**										
No exposure										
Rare	0.009	-0.302	-0.003	7.3		0.011	-0.221	-0.002	7.3	
Frequent	0.012	0.120	0.001	-3.6	3.6	0.024	0.267	0.006	-19.1	-11.8
**Wealth Index**										
Poorest										

Poorer	-0.009	-0.524	0.005	-12.2		0.001	-0.521	0.000	1.4	
Middle	-0.010	-0.090	0.001	-2.3		-0.005	-0.110	0.001	-1.7	
Richer	-0.022	0.346	-0.008	20.0		-0.015	0.364	-0.005	16.4	
Richest	-0.040	0.778	-0.031	79.7	85.2	-0.028	0.806	-0.023	67.9	84.1
**Caste**										
SC/ST										
Non-SC/ST	-0.044	0.089	-0.004	10.1	10.1	-0.033	0.086	-0.003	8.5	8.5
**Religion**										
Hindu										
Non-Hindu	-0.003	0.177	-0.001	1.3	1.3	-0.006	0.110	-0.001	2.1	2.1
**Residence**										
Urban										
Rural	0.008	-0.090	-0.001	1.8	1.8	-0.014	-0.067	0.001	-2.8	-2.8
**States**										
Uttar Pradesh										
Bihar	-0.026	-0.178	0.005	-11.7	-11.7	0.006	-0.182	-0.001	3.1	3.1
**Calculated CI**			-0.039	100.0				-0.033	100.0	
**Actual CI**			-0.161					-0.090		
**Residual**			-0.122					-0.057		

CI: Concentration Index; SC/ST: Scheduled Caste/Scheduled Tribe; %: Percentage

Table-4c Estimates of decomposition analysis for contribution of various explanatory variables for co-existence of thinness and stunting among adolescents aged 10-19 years

**Background characteristics**	**Adolescent boys**					**Adolescent girls**				
	**Elasticity**	**CI**	**Absolute contribution**	**% contribution**		**Elasticity**	**CI**	**Absolute contribution**	**% contribution**	
**Age (years)**										
Early adolescents (10-14)										
Late adolescents (15-19)	0.019	0.088	0.002	-7.3	-7.3	-0.019	0.016	0.000	2.7	2.7
**Educational status (years)**										
No schooling										
1-7	0.036	-0.092	-0.003	14.5		-0.004	-0.092	0.000	-3.2	

8-9	-0.001	0.064	0.000	0.2		-0.003	0.036	0.000	0.9	
10 and above	-0.009	0.269	-0.002	11.0	25.7	-0.003	0.272	-0.001	6.5	4.2
**Working status**										
No										
Yes	-0.012	-0.154	0.002	-8.4	-8.4	-0.001	-0.260	0.000	-2.6	-2.6
**Media exposure**										
No exposure										
Rare	0.011	-0.305	-0.003	14.4		-0.005	-0.221	0.001	-9.3	
Frequent	0.004	0.120	0.000	-2.0	12.4	-0.012	0.267	-0.003	28.2	18.9
**Wealth Index**										
Poorest										
Poorer	-0.008	-0.527	0.004	-18.5		-0.004	-0.521	0.002	-21.0	
Middle	-0.008	-0.092	0.001	-3.1		-0.001	-0.110	0.000	-1.4	
Richer	-0.017	0.347	-0.006	25.4		-0.009	0.364	-0.003	31.1	
Richest	-0.022	0.778	-0.017	75.6	79.4	-0.008	0.806	-0.007	62.1	70.8
**Caste**										
SC/ST										
Non-SC/ST	-0.007	0.088	-0.001	2.8	2.8	-0.016	0.086	-0.001	12.8	12.8
**Religion**										
Hindu										
Non-Hindu	-0.002	0.175	0.000	1.7	1.7	0.002	0.110	0.000	-1.5	-1.5
**Residence**										
Urban										
Rural	0.008	-0.091	-0.001	3.2	3.2	0.005	-0.067	0.000	3.2	3.2
**States**										
Uttar Pradesh										
Bihar	-0.012	-0.175	0.002	-9.4	-9.4	-0.005	-0.182	0.001	-8.4	-8.4
**Calculated CI**			-0.023	100.0				-0.011	100.0	
**Actual CI**			-0.252					-0.173		
**Residual**			-0.229							

CI: Concentration Index; SC/ST: Scheduled Caste/Scheduled Tribe; %: Percentage

Discussion:

Along with measuring prevalence and associated factors of thinness and stunting, this article also examined socio-economic inequality for thinness and stunting among adolescent boys and girls in two economically backward states of India, namely, Uttar Pradesh and Bihar. Furthermore, this study also focuses on the co-existence of both, i.e., thinness and stunting. To the best of our knowledge, this study is among the first in the field to examine thinness and stunting among adolescent boys and girls separately for two of the economically backward and populous states of India. Mondal and Sen (2010) also examined thinness and stunting among adolescent girls and boys; however, their study was limited to rural areas with minimal sample size [9]. Few other studies also examined thinness and stunting among adolescents. However, most of them were based on primary sample data, which was significantly less in number as compared to this study and did not examine the co-existence of thinness and stunting [18,24]. Few other studies also examined thinness [25,26] and stunting [27] among adolescents separately, however, adopting an approach of examining the co-existence of thinness and stunting among adolescent boys and girls makes this study different from previously available literature in various Indian settings.

The prevalence of thinness was higher among adolescent boys than in adolescent girls. Previously available studies also noticed a higher level of thinness among adolescent boys than in adolescent girls [9]. The prevalence of thinness was found to be 25.8 percent among adolescent boys and 13.1 percent among adolescent girls. Previous other studies noted a higher level of prevalence of thinness among girls and boys than in this study in different settings in India [18,28]. In a multi-country analysis, Candler et al. (2017) noted a somewhat similar prevalence of thinness among adolescent Indian girls [29]. Furthermore, the prevalence of stunting was higher among adolescent girls (39.3 %) than in adolescent boys (25.6 %). Previous studies also noticed the same trends where a higher prevalence of stunting was noticed in adolescent girls than in adolescent boys in various other settings of India [1,9]. Bhargava et al. (2020), in their study on 15-19 years of adolescents in India, found the prevalence of stunting to be 32.2 percent among boys and 34.4 percent among girls [1]. The co-existence of thinness and stunting is another prominent issue, and the study noted a higher prevalence of co-existence of thinness and stunting among adolescent boys than in adolescent girls. The unavailability of literature related to the co-existence of thinness and stunting limits our understanding of the issue in various Indian settings.

Stunting was higher among late adolescents (15-19 years) than early adolescents (10-14 years) and was more severe among late adolescent girls than their counterparts. The above finding is concordant with the previously available literature [9,30]. However, studies in other settings found different results [13]. Stunting was severe among girls and could be attributed to their lower nutritional status than boys [8,31]. Results significantly noted that the odds of thinness among late adolescent girls were lower than the odds of thinness among early adolescent girls, and the same has been corroborated with the findings of Gebregyorgis, Tadesse, & Atenafu [13]. Furthermore, a study in rural Indian setting also noted the same result [32]. The thinness among early adolescents generally found to be higher than in late adolescents, possible due to increased growth spurt in the early adolescent stage as compared to the late adolescent stage [13]. Baliga, Naik, & Mallapur (2014), in their community-based rural study in Belgaum, Karnataka, noted that calorie intake deficiency was higher among early adolescent girls as compared to late-adolescent girls, which could be another possible reason of higher thinness among early adolescent girls than their counterparts [32].

Education among adolescents is another important factor in determining stunting and thinness among them. Results noticed that thinness and stunting among adolescent boys and girls were lower among those who had ten and above years of education than their counterparts. Previously available literature also noticed the protective effect of education on the occurrence of stunting and thinness among adolescents [33-35]. Adolescents who attain higher education may receive awareness about nutrition from academic courses that may further improve their nutritional status for them [36]. Few studies have also noted the importance of higher education among the head of the families and parents in reducing the stunting and thinness among adolescents [27,28,30]. Low education among parents lower their decision-making and reduce the contribution to the total family income, which places the family at the risk of not meeting nutritional needs, thus placing a nutritional burden on adolescents [13]. Furthermore, education among mothers promotes personal hygiene and curative health care in households, which leads to better nutritional outcomes among adolescents [30].

The working status of adolescent boys was also found to be significantly affecting thinness among them. Results found that working status improves the odds of being stunted among adolescent boys, and the odds of co-existence of stunting and thinness were also low among working adolescent boys. Working status among adolescents may ensure income, which can further be used to improve nutritional intake. Adolescents from the richest wealth quintile were found to be having lower odds of stunting and thinness. Wealth index has also been noticed as

one of the most significant contributors to the socio-economic inequality in the prevalence of stunting, thinness, and co-existence of both among adolescents. Moreover, results from the concentration curve also confirmed that stunting and thinness are concentrated among adolescents in poor households. The previously available literature is in line with this study in finding the protective feature of increasing household's wealth index or household's monthly income on stunting and thinness among adolescents [3, 30, 37]. Previous studies have linked higher household wealth to quality food and better utilization of health-care services that may be attributed to the lower odds of stunting and thinness among adolescents [38-40].

Due to the nature of the study design, it was not possible to establish a cause-effect relationship. The findings from this study may not be generalizable in other Indian settings because data were available for only two of the total 36 states and union territories of India. Despite the above limitations, this study has certain strengths too. The study involved a large sample size, and information on stunting and thinness were collected using standard tools. This study has examined the co-existence of stunting and thinness among adolescents and paved the way for future research that may aim to strengthen the findings.

Conclusion:

The findings from this study highlighted several important issues related to adolescent's stunting and thinness. The prevalence of thinness was higher among adolescent boys, and that of stunting was higher among adolescent girls. Furthermore, the co-existence of both stunting and thinness was higher among adolescent boys than in adolescent girls. Results also noticed that pro-poor inequality in stunting, thinness, and co-existence of both was higher for adolescent boys than in adolescent girls; it means stunting, thinness, and co-existence of both was more among adolescent boys belonging to poor households. Results from decomposition analysis revealed that the wealth index of the households explained most of the inequality in stunting, thinness, and co-existence of both among adolescents. At first, further research is needed to confirm factors associated with the co-existence of stunting and thinness among adolescents. These results provide an understanding that stunting and thinness is a significant public health concern among adolescents, and there is a need to tackle the issue comprehensively. By tackling the issue comprehensively, we mean that the state government of Uttar Pradesh and Bihar shall screen, assess, and monitor the nutritional status of adolescent boys and girls. Education is an important factor, and ensuring education without dropout at all levels may be a useful step in reducing stunting and thinness among adolescents. The

interventions shall focus towards both boys as well as girl adolescents, and particular emphasis should be given to adolescents who belonged to poor households. Also, efforts should be taken by stakeholders to increase family wealth status [41].

Acknowledgement: Not applicable

Funding: This research did not receive any specific grant from funding agencies in the public, commercial, or not-for-profit sectors.

Declaration of Interests: None

Author's Contribution:

Conceptualization: Pradeep Kumar, Shobhit Srivastava, Shekhar Chauhan, Ratna Patel

Formal Analysis: Pradeep Kumar, Shobhit Srivastava

Investigation: Pradeep Kumar, Shobhit Srivastava, Preeti Dhillon

Methodology: Pradeep Kumar, Shobhit Srivastava

Supervision: Preeti Dhillon

Writing Original Draft: Shekhar Chauhan, Strong Pillar Marbaniang, Ratna Patel

Revising and Finalizing the draft manuscript: Pradeep Kumar, Strong Pillar Marbaniang, Shobhit Srivastava, Shekhar Chauhan, Ratna Patel, Preeti Dhillon

References:

1. Bhargava M, Bhargava A, Ghate SD, Rao RS. Nutritional status of Indian adolescents (15-19 years) from National Family Health Surveys 3 and 4: Revised estimates using WHO 2007 Growth reference. PloS one. 2020 Jun 22;15(6):e0234570. https://doi.org/10.1371/journal.pone.0234570
2. UNICEF. Adolescent demographics [WWW Document]. UNICEF. 2019. URL https://data.unicef.org/topic/adolescents/demographics/ (accessed 9.16.20).
3. Darling AM, Fawzi WW, Barik A, Chowdhury A, Rai RK. Double burden of malnutrition among adolescents in rural West Bengal, India. Nutrition. 2020 Mar 19:110809. https://doi.org/10.1016/j.nut.2020.110809
4. Engidaw MT, Gebremariam AD. Prevalence and associated factors of stunting and thinness among adolescent Somalian refugee girls living in eastern Somali refugee camps, Somali regional state, Southeast Ethiopia. Conflict and health. 2019 Dec 1;13(1):17. https://doi.org/10.1186/s13031-019-0203-3
5. World Health Organization (WHO). Nutrition in adolescence : issues and challenges for the health sector : issues in adolescent health and development, World Health Organization. Geneva, Switzerland. 2005.
6. Lifshitz F, Tarim O, Smith MM. Nutrition in adolescence. Endocrinology and metabolism clinics of North America. 1993 Sep 1;22(3):673-83. https://doi.org/10.1016/S0889-8529(18)30157-9
7. World Health Organization. Physical status: The use of and interpretation of anthropometry, Report of a WHO Expert Committee. World Health Organization; 1995.
8. Medhi GK, Hazarika NC, Mahanta J. Nutritional status of adolescents among tea garden workers. The Indian Journal of Pediatrics. 2007 Apr 1;74(4):343-7. https://doi.org/10.1007/s12098-007-0057-3
9. Mondal N, Sen J. Prevalence of stunting and thinness among rural adolescents of Darjeeling district, West Bengal, India. Italian journal of public health. 2010;7(1). https://doi.org/10.2427/5747
10. Müller O, Krawinkel M. Malnutrition and health in developing countries. Cmaj. 2005 Aug 2;173(3):279-86. https://doi.org/10.1503/cmaj.050342
11. World Health Organization (WHO), n.d. Maternal, Newborn, Child & Adolescent Health: Data portal [WWW Document]. URL https://www.who.int/data/maternal-

newborn-child-adolescent/indicator-explorer-new/mca/prevalence-of-thinness-among-adolescents-aged-10-19-years-(bmi--2-standard-deviations-below-the-median-(crude-estimate)) (accessed 9.16.20).

12. Sethi V, Gupta N, Pedgaonkar S, Saraswat A, Singh KD, Rahman HU, de Wagt A, Unisa S. Mid-upper arm circumference cut-offs for screening thinness and severe thinness in Indian adolescent girls aged 10–19 years in field settings. Public health nutrition. 2019 Aug;22(12):2189-99. https://doi.org/10.1017/S1368980019000594
13. Gebregyorgis T, Tadesse T, Atenafu A. Prevalence of thinness and stunting and associated factors among adolescent school girls in Adwa Town, North Ethiopia. International journal of food science. 2016 May 16;2016. https://doi.org/10.1155/2016/8323982
14. Dobner J, Kaser S. Body mass index and the risk of infection-from underweight to obesity. Clinical Microbiology and Infection. 2018 Jan 1;24(1):24-8. https://doi.org/10.1016/j.cmi.2017.02.013
15. Victora CG, Adair L, Fall C, Hallal PC, Martorell R, Richter L, Sachdev HS, Maternal and Child Undernutrition Study Group. Maternal and child undernutrition: consequences for adult health and human capital. The lancet. 2008 Jan 26;371(9609):340-57. https://doi.org/10.1016/S0140-6736(07)61692-4
16. MöLler B, Lindmark G. Short stature: an obstetric risk factor? A comparison of two villages in Tanzania. Acta obstetricia et gynecologica Scandinavica. 1997 May;76(5):394-7. https://doi.org/10.3109/00016349709047817
17. Konwar P, Vyas N, Hossain SS, Gore MN, Choudhury M. Nutritional status of adolescent girls belonging to the tea garden estates of Sivasagar district, Assam, India. Indian journal of community medicine: official publication of Indian Association of Preventive & Social Medicine. 2019 Jul;44(3):238. https://doi.org/10.4103/ijcm.IJCM_357_18
18. Roy S, Barman S, Mondal N, Sen J. Prevalence of stunting and thinness among adolescent girls belonging to the rajbanshi population of West Bengal, India. Journal of Nepal Paediatric Society. 2016 Dec 31;36(2):147-55. https://doi.org/10.3126/jnps.v36i2.14535
19. Santhya, K.G., Acharya, R., Pandey, N., Singh, S.K., Rampal, S., Zavier, A.J.F., Gupta, A.K. Understanding the lives of adolescents and young adults (UDAYA) in Bihar and Uttar Pradesh, India, New Delhi: Population Council. 2017.

20. Onis MD, Onyango AW, Borghi E, Siyam A, Nishida C, Siekmann J. Development of a WHO growth reference for school-aged children and adolescents. Bulletin of the World health Organization. 2007;85:660-7.
21. WHO. Anthroplus for personal computers manual: Software for assessing growth of the world's children and adolescents. Department of Nutrition for Health and Development. Geneva: WHO; 2009.
22. O'donnell O, Van Doorslaer E, Wagstaff A, Lindelow M. Analyzing health equity using household survey data: a guide to techniques and their implementation. The World Bank; 2007 Oct 27.
23. Wagstaff A, Paci P, Van Doorslaer E. On the measurement of inequalities in health. Social science & medicine. 1991 Jan 1;33(5):545-57. https://doi.org/https://doi.org/10.1016/0277-9536(91)90212-U
24. Maiti S, De D CK, Jana K, Ghosh D, Paul S. Prevalence of stunting and thinness among early adolescent school girls of Paschim Medinipur district, West Bengal. Int J Biol Med Res. 2011;2(3):781-3.
25. Deshmukh PR, Gupta SS, Bharambe MS, Dongre AR, Maliye C, Kaur S, Garg BS. Nutritional status of adolescents in rural Wardha. The Indian Journal of Pediatrics. 2006 Feb 1;73(2):139-41. https://doi.org/10.1007/BF02820204
26. Mondal N. Thinness as major underlying problem among adolescents of Northeast India. Journal of Nepal Paediatric Society. 2014 Mar 24;34(1):39-47. https://doi.org/10.3126/jnps.v34i1.8922
27. Rengma MS, Bose K, Mondal N. Socio-economic and demographic correlates of stunting among adolescents of Assam, North-east India. AnthropologicAl review. 2016 Dec 1;79(4):409-25.
28. Pal A, Pari AK, Sinha A, Dhara PC. Prevalence of undernutrition and associated factors: A cross-sectional study among rural adolescents in West Bengal, India. International Journal of Pediatrics and Adolescent Medicine. 2017 Mar 1;4(1):9-18. https://doi.org/10.1016/j.ijpam.2016.08.009
29. Candler T, Costa S, Heys M, Costello A, Viner RM. Prevalence of thinness in adolescent girls in low-and middle-income countries and associations with wealth, food security, and inequality. Journal of Adolescent Health. 2017 Apr 1;60(4):447-54. https://doi.org/10.1016/j.jadohealth.2016.11.003

30. Gagebo DD, Kerbo AA, Thangavel T. Undernutrition and Associated Factors among Adolescent Girls in Damot Sore District, Southern Ethiopia. Journal of Nutrition and Metabolism. 2020 Jun 25;2020. https://doi.org/10.1155/2020/5083140
31. MalhotraPhD A, Passi SJ. Diet quality and nutritional status of rural adolescent girl beneficiaries of ICDS in north India. Asia Pac J Clin Nutr. 2007;16(1):8-16.
32. Baliga SS, Naik VA, Mallapur MD. Nutritional status of adolescent girls residing in rural area: A community-based cross-sectional study. Journal of the Scientific Society. 2014 Jan 1;41(1):22. https://doi.org/10.4103/0974-5009.126712
33. Mengesha DK, Prasad RP, Asres DT. Prevalence and Associated Factors of Thinness among Adolescent Students in Finote Selam Town, Northwest Ethiopia. The Scientific World Journal. 2020 Jun 2;2020. https://doi.org/10.1155/2020/9170301
34. Berhe K, Gebremariam G. Magnitude and associated factors of undernutrition (underweight and stunting) among school adolescent girls in Hawzen Woreda (District), Tigray regional state, Northern Ethiopia: Cross-sectional study. BMC Research Notes. 2020 Dec 1;13(1):59. https://doi.org/10.1186/s13104-020-4926-4
35. Ismail A, Darling AM, Mosha D, Fawzi W, Sudfeld C, Sando MM, Abdallah Noor R, Charles J, Vuai S. Prevalence and risk factors associated with malnutrition among adolescents in rural Tanzania. Tropical Medicine & International Health. 2020 Jan;25(1):89-100. https://doi.org/10.1111/tmi.13331
36. Bwalya BB. Nutritional status among female adolescents aged (15–19 years) in Zambia: why it matters. Horizon. 2015 Nov;1(1):001-7.
37. Van Tuijl CJ, Madjdian DS, Bras H, Chalise B. Sociocultural and economic determinants of stunting and thinness among adolescent boys and girls in Nepal. Journal of Biosocial Science. 2020 Jul 8:1-26. https://doi.org/10.1017/S0021932020000358
38. Bashir MK, Schilizzi S. Determinants of rural household food security: a comparative analysis of African and Asian studies. Journal of the Science of Food and Agriculture. 2013 Apr;93(6):1251-8. https://doi.org/10.1002/jsfa.6038
39. Sreeramareddy CT, Ramakrishnareddy N, Subramaniam M. Association between household food access insecurity and nutritional status indicators among children aged< 5 years in Nepal: results from a national, cross-sectional household survey. Public health nutrition. 2015 Nov;18(16):2906-14. https://doi.org/10.1017/S1368980014002729

40. Nepali S, Simkhada P, Davies I. Trends and inequalities in stunting in Nepal: a secondary data analysis of four Nepal demographic health surveys from 2001 to 2016. BMC nutrition. 2019 Dec 1;5(1):19. https://doi.org/10.1186/s40795-019-0283-x
41. Tariku EZ, Abebe GA, Melketsedik ZA, Gutema BT. Prevalence and factors associated with stunting and thinness among school-age children in Arba Minch Health and Demographic Surveillance Site, Southern Ethiopia. PloS one. 2018 Nov 2;13(11):e0206659. https://doi.org/10.1371/journal.pone.0206659
