# Supplementary material for: Associated factors and socio-economic inequality in the prevalence of thinness and stunting among adolescent boys and girls in Uttar Pradesh and Bihar, India
Source: PLoS One. 2021 Feb 24;16(2):e0247526. doi: 10.1371/journal.pone.0247526 (PMC7904204; doi:10.1371/journal.pone.0247526)
Supplement: Attachment — Submitted filename: Reviewers comment.docx [file pone.0247526.s001.docx]

**Reviewer comment**

**Associated factors and socio-economic inequality in the prevalence of thinness and**

**stunting among adolescent boys and girls in Uttar Pradesh and Bihar, India**

The reviewer must acknowledge the effort of the authors made by writing this article. Over all written and explained nicely.

- Abstract: Structurally written.
- Introduction: Looks okay
- Methods:
- The basis of sample size 20594 need to be clarified a bit. Also, how the sub samples from each stratum was selected that need to be highlighted
- Was there any inclusion or exclusion criteria at the time of selection of those subjects?
- Though the following things mention elsewhere by the authors but still a little description of the following 3 things will help to understand better

a) Multi stage systematic sampling design need to be illustrated in a figure
b) Survey tools also need to be mentioned
c) A little description of actual data collection process also requires

- Results:
- Why girls are more vulnerable to chronic malnutrition while boys are acutely malnourished? Though more boys suffer from both malnutrition?
- How do you explain that total percentage of thinness and stunting among boys is little lower than total percentage of thinness and stunting among girls; but still co-existence of both is higher among boys?
